# Cryptosporidium and giardia species in newly and previously habituated gorillas and nearby water sources in Bwindi Impenetrable National Park, Uganda

**DOI:** 10.11604/pamj.2019.34.112.19288

**Published:** 2019-10-25

**Authors:** Gizamba Jacob Mugoya, Celsus Sente, Samuel Nambile Cumber, Kabanda Taseera, Claude Ngwayu Nkfusai, Catherine Atuhaire

**Affiliations:** 1 College of Veterinary Medicine, Animal Resources and Biosecurity, School of Veterinary Medicine and Animal Resources, Department of Wildlife and Aquatic Animal Resources, Makerere University, Kampala, Uganda; 2 Section for Epidemiology and Social Medicine, Department of Public Health, Institute of Medicine (EPSO), The Sahlgrenska Academy at University of Gothenburg, Gothenburg, Sweden; 3 Faculty of Health Sciences, University of the Free State, Bloemfontein, South Africa; 4 School of Health Systems and Public Health, Faculty of Health Sciences, University of Pretoria Private Bag X323, Gezina, Pretoria, South Africa; 5 Faculty of Medicine, Department of Microbiology, Mbarara University of Science and Technology, Mbarara, Uganda; 6 Department of Microbiology and Parasitology, Faculty of Science, University of Buea, Buea, Cameroon; 7 Faculty of Medicine, Department of Nursing, Mbarara University of Science and Technology, Mbarara, Uganda

**Keywords:** Cryptosporidium, giardia, Newly Habituated Mountain Gorillas, Previously Habituated Mountain Gorillas, water

## Abstract

**Introduction:**

cryptosporidium and giardia are of great one health significance and major cause of protozoan diarrhea in humans and primates; they are found in the faecal matter of animals and humans and also in contaminated water and soil as well. Therefore, we aimed at establishing the prevalence and shedding intensity of faecal Cryptosporidium and giardia in the Newly Habituated Mountain Gorillas (NHMG) and Previously Habituated Mountain Gorillas (PHMG) and in selected water sources within the gorilla home ranges in the month of January 2018.

**Methods:**

we conducted a cross sectional study in the southern sector of Bwindi Impenetrable National Park where a total of 56 faecal samples from both NHMG (34) and PHMG (22) and 30 water samples were purposively collected. Faecal and water samples were transported in a cooler box at 4ºC to Makerere University Parasitology Laboratory for analysis. The samples were analyzed using modified Ziehl-Neelsen technique and Ether concentration method for Cryptosporidium and giardia respectively.

**Results:**

the prevalence of cryptosporidium was established as 13 (59.1%), 15 (44.1%) and 7 (23.3%) in PHMG, NHMG and water respectively. The mean concentration of the oocysts per gram was 222±52.9 in PHMG, 174±41.5 in NHMG and 31±13.2 in water. The prevalence of giardia was 3 (13.6%), 4 (11.8%) and 3 (10%) in PHMG, NHMG and water respectively. The mean concentration of the oocysts per gram was 34±19.9 in PHMG, 25±12.4 in NHMG and 5±2.9 in water. There was no significant difference in both the prevalence of cryptosporidium (p>0.05) and giardia (p>0.05) in the PHMG and NHMG. This indicates that there is high risk of cross infection among the gorillas within the forest sharing similar home ranges.

**Conclusion:**

the park authorities should ensure that procedures for proper waste disposal while in the forest are properly followed, water drawn for drinking from the forest should be avoided. Further research should be carried out to identify whether the strains of the parasites found in water or other animals in the forest are the same with strains in gorilla dung in order to confirm cross infection.

## Introduction

Cryptosporidium and giardia are ubiquitous enteric microscopic protozoon parasites that infects humans, domestic animals and wild animals worldwide [1]. These organisms are the major causes of protozoan diarrhea in humans and other primates [2]; they are found in the faecal matter of animals and humans and also in contaminated water even in soil as well. When ingested by humans, livestock or wildlife in contaminated water, they cause giardiasis (beaver fever) and cryptosporidiosis whose major symptom is diarrhea and leads to considerable morbidity and mortality. Cryptosporidium and giardia from cattle are potential zoonotic pathogens and contact with animals, manure or contaminated water leads to infections in humans. Both organisms cause gastrointestinal diseases and transmission is sustained by both zoonotic and anthropologic cycles [3, 4]. Waterborne out breaks of cryptosporidium and giardia have been reported in the past several decades [3], therefore information on the occurrence of these protozoa in the water sources and nonhuman primates is necessary. Cryptosporidium and giardia have transmission or infective stages that are oocysts and cysts respectively, which are immediately infectious when excreted in faeces and can be transmitted by person to person, or animal to person contact [3]. The oocysts are highly resistant to environmental stress and standard water treatment technologies [5]. The resistant stages are microscopic in size and of low specific gravity, which facilitates their dissemination in the water sources [6].

In Uganda according to [7, 8], Cryptosporidium has been extensively discovered in mountain gorillas but none of these describe the associated risks or the presence and intensity of these organisms in the water sources and the relationship between the occurrence of these organisms in both mountain gorillas and the water sources. Recent studies have documented evidence of cryptosporidium and giardia transmission from wild primates to humans [9] and to livestock via water contamination. There is also a greater recognition of the potential risk of human pathogen transmission to free ranging primates via water sources [10]. Therefore, due to the interaction and sharing of the water sources by humans, wildlife and livestock in Bwindi Impenetrable National Park (BINP) it is vital to investigate the current situation in-terms of prevalence and shedding intensity of faecal cryptosporidium and giardia in newly and previously habituated mountain gorillas in BINP and in selected water sources around the park that are shared by the wildlife and the local communities. The findings will help the park authorities to regulate and properly monitor mountain gorilla health and also enforce evidence based health awareness education to the surrounding communities on the water they use. The study determined the prevalence and shedding intensity of faecal cryptosporidium and giardia species in newly and previously habituated mountain gorillas and selected water sources within their home ranges in BINP, Uganda. 1) To detect the presence and prevalence of faecal cryptosporidium and giardia spp in newly and previously habituated mountain gorillas and selected water sources in BINP. 2) To determine the shedding intensity of the faecal cryptosporidium and giardia in newly and previously habituated mountain gorillas and recovery efficiency of the parasites in water sources.

## Methods

**Study design:** a cross sectional study was conducted where faecal matter of NHMG and PHMG were purposively and conveniently collected to determine the presence, prevalence and intensity of cryptosporidium and giardia spp. Water samples were also collected purposively and conveniently to determine prevalence and recovery efficiency of the parasites in water.

**Study setting:** the study was conducted in Bwindi Impenetrable National Park (BINP) located in Southwestern Uganda on the edge of the Albertine rift valley and it's shared among three districts: Kabale, Kanungu and Kisoro. It covers approximately 321 square kilometers and from its borders it's surrounded by the community land. It harbors a variety of wildlife which includes elephants, mountain gorillas, chimpanzees, and a variety of primates, bird species, butterflies, reptiles and duikers. It is famously known to be a habitat of over 400 critically endangered mountain gorillas which make up to almost half of the entire population worldwide [11]. Around half of the population of the mountain gorillas has been habituated for tourism purposes. The area around the park is densely populated [12], and people are more engaged in mixed farming which involves livestock keeping and crop farming. Animal rearing in this area which is densely populated has increased the risks of transmission of zoonotic diseases and disease causing organisms from humans to animals and also from animals to humans. This is because of sharing the same foraging grounds and water sources around the park. Previously habituated gorilla groups were taken to be those which were habituated and opened for tourism before 2010 and newly habituated gorillas' groups considered were those which had just been opened for tourism or those which were not officially opened for tourism but their habituation process was soon complete.

**Study population:** BINP is a home to fifteen habituated mountains gorillas' groups, four groups were selected from two different home ranges, two of which are Previously Habituated Groups and the other two are Newly Habituated Groups. These groups included Bikingi (22 gorillas) and Kahungye (17 gorillas) from Rushaga tourism zone, Bushaho (10 gorillas) and Nkuringo (13 gorillas) from Nkuringo tourism zone. These groups move around their home ranges and also in the community land which is at the interface of the park foraging and crossing while drinking on different water sources available. This exposes them to risks of being infected with giardia and Cryptosporidium.

**Sample collection:** gorilla faecal sample was collected non-invasively from each accessible gorilla night nest in Bikingi, Bushaho, Kahungye and Nkuringo groups. A total of 56 faecal samples from gorilla nests were collected; 22 in Bikingi, Kahungye group (11 nests), Nkuringo group (11 nests), Bushaho group (12 nests). Some nests were missed because they were too high in trees and some in inaccessible areas. Each faecal sample was physically examined carefully before collection, to ensure whether it belonged to an infant, sub adult, juvenile or adult female or Silverback. Approximately 10 g of faecal sample were collected aseptically from the inner mass of faecal lobe and put in faecal sampling cups containing 10% formalin, stored at 4 degrees Celsius and transported to the parasitology laboratory at the College of Veterinary Medicine, Animal Resources and Biosecurity (COVAB) within 24 hours for analysis. The water samples were collected conveniently and purposively along selected water points and sources within the home ranges of the gorilla groups, from within the park to the outside near community land. Assistance from the rangers who follow the gorilla groups daily was considered, because the rangers know the water spots which could be of importance in this study. In total 30 water samples were collected from the different home ranges of the gorilla groups under study. The water sources from which the water samples were obtained included ponds, streams, swamp and river (R. Kashasha). The water samples were collected in 60 ml sterile sampling cups and all samples were stored in a cooler at 4^o^C while in the field and then the samples were transferred to the fridge at temperature ranging from 4-8ºC. Study variables: the main outcome variable was the prevalence and shedding intensity of cryptosporidium and giardia in the faecal matter and water samples collected. The independent variables included; gorilla group habituation status (previously or newly), gorilla groups, gorilla group composition, year of habituation, water source sampled, and location of water source.

**Laboratory analysis: modified Ziehl-Neelsen technique for cryptosporidium:** a small amount (1 g) of faecal sample was gotten and put on a labeled glass slide to make a smear. The smear was then air-dried and placed on 2 rods over a sink and carbolfuchsin dispensed on the slide smear. A spirit lamp was used to flame the underneath until vapour came off. This was done after every 5 minutes for three times. The stain was then washed off with tap water and acid ethyl alcohol, dispensed on the smear and left for 30-60 seconds. Brilliant green was then dispensed as a counter stain for 2 minutes and excess stain washed off with tap water. The smear was then left to air dry. It was examined under a light microscope with an objective lens of x100 using oil immersion [13]. Oocysts appeared as circular bodies staining red or pink and surrounded by a space or halo. A number of observed oocysts were counted and recorded. For water samples 15mls were first put into a centrifuge tube and centrifuged at 6000rpm for 3 minutes. The supernatant was poured off and the sediment was obtained, a smear was made on the glass slide and the same process was followed as done for the faecal matter.

**Ether concentration method for giardia:** a small amount of faecal matter was put in a beaker, 15ml of sodium acetate formaldehyde (SAF) then added and a mixture was made. The solution was then filtered into another beaker. The filtrate was then transferred into a centrifuge tube and centrifuged at 2000rpm for 1 minute. The supernatant after centrifuge was then poured and 7ml of sodium chloride solution and 2ml of ether were added to the sediment. This was then centrifuged again at 2000rpm for 3 minutes. The supernatant was poured off, and using a dropper, the sediment was picked and put into a glass slide, which was then covered with a cover slip and viewed under a light microscope at an objective lens of x100. The number of cysts were counted and recorded.

**Data analysis:** collected data from questionnaires were entered into separate templates in Excel version 10. The data was verified for completion, cleaned and exported into SPSS v 16.0 for analyses. Descriptive analysis was carried out by calculating the mean, median, standard deviation and frequencies of different variables using SPSS v 16.0. The prevalence rates of cryptosporidium and giardia in both the PHMG and NHMG and water samples were retrospectively calculated. Chi-square test was used to: assess if there is an association between prevalence of cryptosporidium and giardia in both the PHMG and NHMG and recovery efficiency of these protozoans in water sources within their home ranges. Significant level was set at p<0.05.

**Ethical consideration:** this study was approved by the department of Wildlife and Aquatic Animal Resources (WAAR), College of Veterinary Medicine, Animal Resources and Biosecurity, Makerere University. It was further approved by Uganda Wildlife Authority (UWA), which provided a permit to conduct this study in BINP.

## Results

**Composition of different gorilla groups sampled:** four gorilla groups were sampled; Bikingi (22 gorillas), Kahungye group (11 gorillas), Nkuringo group (11 gorillas) and Bushaho group (12 gorillas). The gorilla groups were grouped basing on year of habituation, tourism one where they are found and break down of the composition of each gorilla group is given basing on number of infants, juveniles, sub-adults, adult females and silverbacks in the group (Table 1).

**Table 1:** Composition of the sampled gorilla groups, year of habituation and their habituation status in the two different tourism zones

Tourism zone	Gorilla Groups	Habituation status	Year of habituation	Group composition	
				Age group	Number of individuals
Rushaga	Kahungye	PH	2008-2010	Infant	1
				Juvenile	2
				Sub adult	3
				Adult female	1
				Black back	2
				Silver back	2
				Total	11
	Bikingi	NH	2014-2016	Infant	6
				Juvenile	3
				Sub adult	3
				Adult female	6
				Black back	3
				Silver back	1
				Total	22
Nkuringo	Nkuringo	PH	1998-2004	Infant	1
				Juvenile	1
				Sub adult	2
				Adult female	2
				Black back	3
				Silver back	2
				Total	11
	Bushaho	NH	2014-2016	Infant	2
				Juvenile	3
				Sub adult	2
				Adult female	3
				Black back	1
				Silver back	1
				Total	12

**PH**; Previously Habituated, **NH**; Newly Habituated

**Prevalence of cryptosporidium and giardia spp in the NHMG and PHMG:** a total of 56 faecal samples were collected, 22 were for the PHMG while 34 were for the NHMG from the southern sector of Bwindi Impenetrable National Park. Out of the 22 PHMG 59.1% (13/22) tested positive for cryptosporidium and 13.6% (3/22) tested positive for giardia. The newly habituated gorillas had 44.1% (15/34) positive for cryptosporidium oocysts and 11.8% (4/34) tested positive for giardia (Table 2). The prevalence within each respective gorilla group sampled was 72.2%, 40.9%, 45.5% and 50.0% for cryptosporidium in Kahungye, Bikingi, Nkuringo and Bushaho respectively while 9.1%, 9.1%, 18.2% and 16.7% for giardia respectively (Table 3). There was no difference (X^2^ = 0.274, p>0.05 and X^2^ = 0.836, p>0.05) between the prevalence of cryptosporidium and giardia in the PHMG and NHMG. The chi-square test values between the different gorilla groups sampled showed that there was no significant difference between the prevalence of the two protozoans in the different sampled gorilla groups.

**Table 2:** Overall prevalence of cryptosporidium and giardia in the gorilla groups

	Gorillas	Cryptosporidium		Giardia	
		Frequency (+ves)	% Prevalence	Frequency (+ves)	% Prevalence
	Overall (56)	28	50.0	7	12.5
Habituation status	PHMG (22)	13	59.1	3	13.6
	NHMG (34)	15	44.1	4	11.8
Gorilla groups	Kahungye(11)	8	72.7	1	9.1
	Bikingi (22)	9	40.9	2	9.1
	Nkuringo (11)	5	45.5	2	18.2
	Bushaho (12)	6	50.0	2	16.7

**PHMG;** Previously habituated mountain gorillas, **NHMG;** Newly habituated mountain gorillas

**Table 3:** Prevalence of cryptosporidium and giardia in specific age groups of gorillas sampled

Tourism zone	Gorilla Groups	Group composition		Cryptosporidium		Giardia	
		Age group	Number of individuals	Frequency (+ves)	% Prevalence	Frequency (+ves)	% Prevalence
Rushaga	Kahungye	Infant	1	1	9.1	0	0.0
		Juvenile	2	1	9.1	0	0.0
		Sub adult	3	2	18.2	1	9.1
		Adult female	1	0	0.0	0	0.0
		Black back	2	2	18.2	0	0.0
		Silver back	2	2	18.2	0	0.0
	Bikingi	Infant	6	1	4.5	1	4.5
		Juvenile	3	2	9.1	0	0.0
		Sub adult	3	2	9.1	0	0.0
		Adult female	6	2	9.1	1	4.5
		Black back	3	2	9.1	0	0.0
		Silver back	1	0	0.0	0	0.0
Nkuringo	Nkuringo	Infant	1	0	0.0	0	0.0
		Juvenile	1	0	0.0	0	0.0
		Sub adult	2	2	18.2	0	0.0
		Adult female	2	1	9.1	1	9.1
		Black back	3	1	9.1	1	9.1
		Silver back	2	1	9.1	0	0.0
	Bushaho	Infant	2	0	0.0	1	8.3
		Juvenile	3	1	8.3	1	8.3
		Sub adult	2	1	8.3	0	0.0
		Adult female	3	2	16.7	0	0.0
		Black back	1	1	8.3	0	0.0
		Silver back	1	1	8.3	0	0.0

**Prevalence of cryptosporidium and giardia spp in water:** a total of 30 water samples were collected from the different water sources within the home ranges of the sampled gorilla groups. 23.3% (7/30) of the water samples tested positive for cryptosporidium while 10% (3/30) tested positive of giardia cysts. However, most of the water sources that tested positive were in Rushaga tourism zone with six of the seven water samples that were positive of cryptosporidium and all the three samples positive for giardia also came from Rushaga. Nkuringo tourism zone had only one water source which was positive for cryptosporidium and this was River Kashasha (Table 4).

**Table 4:** Prevalence of cryptosporidium and giardia in water

		Cryptosporidium		Giardia	
	Number of samples	Frequency (+ves)	% prevalence	Frequency (+ves)	% prevalence
overall	30	7	23.3	3	10
Pond	10	2	20	2	20
Swamp	4	3	75	1	25
Stream	14	1	7.1	0	0
River	2	1	50	0	0

**Shedding intensity of cryptosporidium and giardia spp in the NHMG and PHMG and recovery efficiency in water:** the shedding intensity of cryptosporidium and giardia spp in the NHMG was 174±41.5 and 25±12.4 respectively and in the PHMG it was 222±52.9 and 34±19.9 respectively. The shedding intensity was overall higher for cryptosporidium than for giardia in both the PHMG and NHMG; however, the shedding intensity for both cryptosporidium and giardia in previously habituated groups was higher than the shedding intensity in the newly habituated groups. The recovery efficiency of cryptosporidium and giardia in water was 31±13.2 and 5±2.9 respectively (Table 5).

**Table 5:** Shedding intensity of cryptosporidium and giardia and recovery efficiency in water

		Oocysts/Cysts per gram	
		Cryptosporidium (mean±SE)	Giardia (mean±SE)
**Shedding intensity in faecal matter**			
Habituation status	PHMG (22)	222±52.9	34±19.9
	NHMG (34)	174±41.5	25±12.4
Gorilla’s groups	Kahungye (11)	320±80.4	21±20.2
	Bikingi (22)	170±54.7	17±11.9
	Nkuringo (11)	124±58.2	47±35
	Bushaho (12)	182±64.5	40±27.9
**Recovery efficiency in water**			
Water	Overall	31±13.2	5±2.9
	Pond	41±33.3	11±7.9
	Swamp	102±35.0	10±9.3
	Stream	6±5.3	0
	River	19±18.5	0

## Discussion

The primary focus of this study was to get an insight of the prevalence in the PHMG and NHMG and water sources, as well as the possibility of cross infection with protozoan parasites. The results showed that all the gorilla groups sampled both in Rushaga and Nkuringo tourism zone were infected with cryptosporidium and giardia. Most of the water sources sampled that tested positive for cryptosporidium and giardia were located in Rushaga tourism zone, and only samples from R. Kashasha in Nkuringo tested positive for cryptosporidium. The presence of cryptosporidium and giardia in both the gorilla faecal matter and the water sources poses a great public health challenge given the zoonotic potential of the protozoans. The prevalence of cryptosporidium and giardia was higher in PHMG than NHMG. The prevalence of both parasites was higher than that reported by [8]. The current high prevalence may be due increased ecotourism activity and rangers that access the gorillas. A study by [8] showed that staff members who came in contact with gorilla dung did not undertake appropriate precautions, and that not all of the staff members buried their own faecal waste after defecating in the park. Local community members have a habit of not burying their faecal waste after promiscuous defecation in the park, and this is probably one of the leading causes of contamination of water in the forest. The prevalence of cryptosporidium in the individual gorilla groups was high in Kahungye (PHG) at 72.7% concurring with findings of [8] who noted 73% in the same group. The NHG that is Bushaho and Bikingi had 50% and 40.9% respectively, indicating high rate of infestation even among the new gorilla groups. The high prevalence in the individual groups may be attributed to coprophagy [14], where a gorilla feed on its own or another individual gorilla faecal matter. Small home ranges in which the gorillas roam can also result into the observed high infection.

The prevalence of cryptosporidium and giardia in water was 23.3% and 10% respectively and mostly the water sources collected from Rushaga tourism zone were positive for both cryptosporidium and giardia. The contamination of water source could have come from the infected gorilla faecal matter when they defecated directly in the water sources or as a result of indirect contamination from runoff water which carries the parasites into the streams and swamps. Elephant dung could probably be having the oocysts which also contaminate the water sources; this is because some water samples collected from the ponds and swamp where elephants wallow from were positive for both cryptosporidium and giardia [15]. Water contamination with oocysts could also be from staff members who don't bury their own faecal waste after defecating in the park, in addition, the local community people do not bury their faecal waste after promiscuous defecation in the park [8], livestock utilizing water sources that flow to the forest can also be probable source of water contamination. Presence of cryptosporidium in the water sources within the gorilla home ranges poses also a risk of infection to the gorillas [16]. Taking into consideration the prevalence from specific water sources where water samples were obtained: pond (20%), stream (7.1%), river (50%), swamp (75%) for cryptosporidium and pond (20%), stream (0%), river (0%), swamp (25%) for giardia, it's evident that water from the swamp had the highest prevalence of the organisms, probably because water in this area was being contaminated much by the different faecal matter of animal species that drink and wallow from and around the swamp. The observed low prevalence of the organisms in the streams can probably be explained by the continuous flow of the water in the stream, whereby the cryptosporidium oocysts and giardia cysts don't have a conducive environment to multiply [17]. These organisms thrive better in stagnant or slow moving water sources. There was no significant difference between the prevalence of cryptosporidium and giardia in the PHMG and NHMG (p>0.05); this was also true when each gorilla group was compared with the other. This therefore shows high possibility of cross infection between the PHMG and NHMG. Cross transmission can be boosted by the sharing of home ranges which is observed among the gorillas, individuals changing from one group to another and shared water sources. This is in agreement with Nizeyi JB *et al.* [8].

## Conclusion

This should state clearly the main conclusions and provide an explanation of the importance and relevance of the study reported. In general the prevalence of cryptosporidium and giardia is high in the PHMG than in the NHMG, with Rushaga tourism zone having a higher cryptosporidium prevalence than Nkuringo tourism zone, whereas Nkuringo zone had a higher prevalence of giardia. There was no significant difference between the prevalence of cryptosporidium and giardia in the PHMG and NHMG and in all the gorilla groups sampled hence all groups posed equal risks to each other. The prevalence of cryptosporidium in the water bodies was higher than that of giardia and most of the water sources in Rushaga tourism zone tested positive for cryptosporidium and giardia hence posing risk of infection to gorillas, tourists, rangers and the nearby community. Most water sources that tested positive were collected from within the forest, indicating that these water bodies are not safe for use by humans (rangers) or visitors, this could also be showing improper waste disposal by humans while in the forest.

### What is known about this topic

- Cryptosporidium and giardia are ubiquitous enteric microscopic protozoon parasites that infects humans, domestic animals and wildlife worldwide;
- These organisms are the major causes of protozoan diarrhea in humans or other primates and they are found in the faecal matter of animals and humans, contaminated water and soil;
- Bwindi impenetrable National Park is a tourist site in Uganda habited mostly by gorillas on the boarders of Uganda, Rwanda and Congo.

### What this study adds

- The prevalence of cryptosporidium and giardia is high in the PHMG than in the NHMG, with Rushaga tourism zone having a higher cryptosporidium prevalence than Nkuringo tourism zone, whereas Nkuringo zone had a higher prevalence of giardia;
- The prevalence of cryptosporidium in the water bodies was higher than that of giardia and most of the water sources in Rushaga tourism zone tested positive for cryptosporidium and giardia hence posing risk of infection to gorillas, tourists, rangers and the nearby community;
- Most water sources that tested positive were collected from within the forest, indicating that these water bodies are not safe for use by humans (rangers) or visitors, this could also be showing improper waste disposal by humans while in the forest.

## References

[1] Savioli L, Smith H, Thompson A (2006). Giardia and cryptosporidium join the 'neglected diseases initiative'. Trends Parasitol.

[2] Cacciò SM, Thompson RCA, McLauchlin J, Smith HV (2005). Unravelling cryptosporidium and giardia epidemiology. Trends Parasitol.

[3] Xiao L, Fayer R (2008). Molecular characterisation of species and genotypes of cryptosporidium and giardia and assessment of zoonotic transmission. Int J Parasitol.

[4] Cacciò SM, Ryan U (2008). Molecular epidemiology of giardiasis. Mol Biochem Parasitol.

[5] DuPont HL, Chappell CL, Sterling CR, Okhuysen PC, Rose JB, Jakubowski W (1995). The infectivity of cryptosporidium parvum in healthy volunteers. N Engl J Med.

[6] Lobo ML, Xiao L, Antunes F, Matos O (2009). Occurrence of cryptosporidium and giardia genotypes and subtypes in raw and treated water in Portugal. Lett Appl Microbiol.

[7] Mwebe RA (1998). Survey on cryptosporidium prevalence within the mountain Gorilla population (Gorilla Gorilla beringei) of the Bwindi Impenetrable National Park in South-western Uganda.

[8] Nizeyi JB, Mwebe R, Nanteza A, Cranfield MR, Kalema GRNN, Graczyk TK (1999). Cryptosporidium sp. and giardia sp. infections in mountain Gorillas (Gorilla Gorilla beringei) of the Bwindi Impenetrable National Park, Uganda. J Parasitol.

[9] Calvignac-Spencer S, Leendertz SAJ, Gillespie TR, Leendertz FH (2012). Wild great apes as sentinels and sources of infectious disease. Clin Microbiol Infect.

[10] Köndgen S, Kühl H, N'Goran PK, Walsh PD, Schenk S, Ernst N (2008). Pandemic human viruses cause decline of endangered great apes. Curr Biol.

[11] Makombo J, Authority UW (2018). Wealth creation through conservation: Bwindi Impenetrable and Rwenzori Mountains National Parks, Uganda1.

[12] Harris D, Joshi A, Khan PA, Gothkar P, Sodhi PS (1999). On-farm seed priming in semi-arid agriculture: development and evaluation in maize, rice and chickpea in India using participatory methods. Exp Agric.

[13] Kaufmann J (1996). Parasites of cattle. Parasitic Infections of Domestic Animals.

[14] Graczyk TK, Fayer R, Cranfield MR (1997). Zoonotic transmission of cryptosporidium parvum: Implications for water-borne cryptosporidiosis. Parasitol Today.

[15] Samra NA, Jori F, Samie A, Thompson P (2011). The prevalence of cryptosporidium spp. oocysts in wild mammals in the Kruger National Park, South Africa. Vet Parasitol.

[16] Nizeyi JB, Sebunya D, Dasilva AJ, Cranfield MR, Pieniazek NJ, Graczyk TK (2002). Cryptosporidiosis in people sharing habitats with free-ranging mountain Gorillas (Gorilla Gorilla beringei), Uganda. Am J Trop Med Hyg.

[17] Ithoi I (2009). Occurrence of giardia and cryptosporidium (oo) cysts in the river water of two recreational areas in Selangor, Malaysia. Trop Biomed.

